# A Probabilistic Model of RNA Conformational Space

**DOI:** 10.1371/journal.pcbi.1000406

**Published:** 2009-06-19

**Authors:** Jes Frellsen, Ida Moltke, Martin Thiim, Kanti V. Mardia, Jesper Ferkinghoff-Borg, Thomas Hamelryck

**Affiliations:** 1 The Bioinformatics Center, Department of Biology, University of Copenhagen, Copenhagen, Denmark; 2 Department of Statistics, University of Leeds, Leeds, United Kingdom; 3 DTU Elektro, Technical University of Denmark, Lyngby, Denmark; Wellcome Trust Sanger Institute, United Kingdom

## Abstract

The increasing importance of non-coding RNA in biology and medicine has led to a growing interest in the problem of RNA 3-D structure prediction. As is the case for proteins, RNA 3-D structure prediction methods require two key ingredients: an accurate energy function and a conformational sampling procedure. Both are only partly solved problems. Here, we focus on the problem of conformational sampling. The current state of the art solution is based on fragment assembly methods, which construct plausible conformations by stringing together short fragments obtained from experimental structures. However, the discrete nature of the fragments necessitates the use of carefully tuned, unphysical energy functions, and their non-probabilistic nature impairs unbiased sampling. We offer a solution to the sampling problem that removes these important limitations: a probabilistic model of RNA structure that allows efficient sampling of RNA conformations in continuous space, and with associated probabilities. We show that the model captures several key features of RNA structure, such as its rotameric nature and the distribution of the helix lengths. Furthermore, the model readily generates native-like 3-D conformations for 9 out of 10 test structures, solely using coarse-grained base-pairing information. In conclusion, the method provides a theoretical and practical solution for a major bottleneck on the way to routine prediction and simulation of RNA structure and dynamics in atomic detail.

## Author Summary

The importance of RNA in biology and medicine has increased immensely over the last several years, due to the discovery of a wide range of important biological processes that are under the guidance of non-coding RNA. As is the case with proteins, the function of an RNA molecule is encoded in its three-dimensional (3-D) structure, which in turn is determined by the molecule's sequence. Therefore, interest in the computational prediction of the 3-D structure of RNA from sequence is great. One of the main bottlenecks in routine prediction and simulation of RNA structure and dynamics is sampling, the efficient generation of RNA-like conformations, ideally in a mathematically and physically sound way. Current methods require the use of unphysical energy functions to amend the shortcomings of the sampling procedure. We have developed a mathematical model that describes RNA's conformational space in atomic detail, without the shortcomings of other sampling methods. As an illustration of its potential, we describe a simple yet efficient method to sample conformations that are compatible with a given secondary structure. An implementation of the sampling method, called BARNACLE, is freely available.

## Introduction

Non-coding RNA is of crucial importance for the functioning of the living cell, where it plays key catalytic, regulatory and structural roles [1],[2]. Understanding the exact mechanisms behind these functions is therefore of great importance for both biology and medicine. In many cases, this understanding requires knowledge of RNA structure in atomic detail. However, determining the structure of an RNA molecule experimentally is typically a time consuming, expensive and difficult task [3]. Therefore, algorithms for RNA structure prediction have attracted much interest, initially with the main focus on predicting secondary structure. Many noticeable advances have been made in the area of secondary structure prediction; most recently the introduction of statistical sampling had an important impact [3]–[5].

In the past years, an increasing number of relevant structures have become available, and much progress has been made in the understanding of the three dimensional (3-D) structure of RNA. The conformational space of RNA has been analyzed using methods inspired by the Ramachandran plot for proteins [6],[7], the RNA base pair interactions have been accurately classified [8], and the conformational space of the RNA backbone has been clustered into discrete recurring conformations [6], [9]–[11]. These new insights have led to several useful tools for modeling RNA 3-D structure [3],[12] and significant advances in atomic resolution prediction have recently been reported [13],[14].

However, routine prediction of RNA 3-D structure still remains an important open problem, and with the growing gap between the number of known sequences and determined structures, the problem is becoming more and more pronounced. The two key ingredients in algorithms for RNA 3-D structure prediction, namely an accurate energy function and a conformational sampling procedure [14], are both only partly solved problems. Here, we focus on the latter problem.

The current state of the art in RNA conformational sampling is based on fragment assembly methods, which construct plausible conformations by stringing together short fragments obtained from experimental structures. These methods have led to numerous important breakthroughs in the related fields of protein and RNA 3-D structure prediction in the last ten years [13]–[15]. Nonetheless, fragment assembly methods are not a panacea. One of the problems associated with these methods is that they inherently discretize the continuous conformational space, and hence do not cover all relevant conformations [14]. This is problematic since the resolution of the conformational search procedure imposes limits on the energy function; the use of fine-grained energy terms requires continuous adjustments to the RNA's dihedral degrees of freedom, which fragment assembly methods cannot provide [14]. In other words, the shortcomings of the conformational sampling method need to be counteracted by tweaking the energy function. Furthermore, full conformational detail is of great importance for a complete understanding of RNA catalysis, binding [9] and dynamics [16].

Another fundamental problem with fragment assembly methods is their non-probabilistic nature, which makes their rigorous use in the framework of statistical physics problematic. Particularly, it is currently impossible to ensure unbiased sampling (which requires the property of detailed balance [17]) in a Markov chain Monte Carlo (MCMC) framework using fragment assembly as a proposal function [18]. In other words, using a fragment library implies adding an inherently unknown additional term to the energy function [18]. This means that the unbiased simulation of the dynamics of an RNA molecule under the control of an all-atom empirical forcefield using fragment assembly methods is currently impossible.

For these reasons we have developed a new solution to the conformational sampling problem: a probabilistic model, called BARNACLE, that describes RNA structure in a natural, continuous space. BARNACLE makes it possible to efficiently sample 3-D conformations that are RNA-like on a short length scale. Such a model can be used purely as a proposal distribution, but also as an energy term enforcing realistic local conformations. Imposing favorable long range interactions, such as hydrogen bonding between the bases, lies outside the scope of such a local model and is the task of a global energy function.

BARNACLE combines a dynamic Bayesian network (DBN) [19], which suits the sequential nature of the RNA molecule, with directional statistics, a branch of statistics that is concerned with the representation of angular data. The model is not only computationally attractive, but can also be rigorously interpreted in the language of statistical physics [20],[21], making it attractive from a theoretical viewpoint as well.

This approach is conceptually related to the probabilistic models of protein structure recently proposed by our group [20],[21]. However, the model presented here is clearly far from a trivial extension, as an RNA molecule has many more degrees of freedom than a protein; in the RNA backbone alone, there are 11 angles per residue [22], as opposed to two in proteins. These many degrees of freedom combined with the limited number of experimentally determined RNA structures [23] make this a particularly challenging statistical task for which a very different strategy was required. In particular, the approach we used for proteins would in the case of RNA require the use of a probability density function on the 7-dimensional hypertorus, which poses a serious statistical and computational obstacle.

Below, we describe the probabilistic model in detail, and show that it captures the crucial aspects of local RNA structure. We also demonstrate its usefulness in the context of RNA 3-D prediction, and end with an outlook on possible applications.

## Results

In this section, we first briefly explain the parameterization of RNA 3-D structure, then describe the probabilistic model and finally present an evaluation of its performance in various contexts.

### Parameterization of RNA 3-D structure

**Figure 1:**
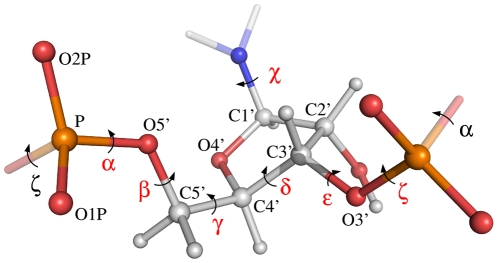
Ball-and-stick representation of an RNA fragment. The seven relevant dihedral angles in the central nucleotide (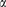 to 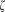) are indicated with red labels. Each label is placed on the central bond of the four consecutive atoms that define the dihedral angle. The 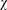 angle describes the rotation of the base relative to the RNA backbone, while the six other angles define the course of the backbone. All atoms in the central nucleotide are labeled and colored according to atom type (oxygen: red, phosphor: yellow, nitrogen: blue and carbon/hydrogen: grey). For clarity, the base is only partly shown.

Before we can formulate a probabilistic model, we need a mathematical parameterization of RNA 3-D structure. For each residue in an RNA molecule, the parameterization consists of the base type (A, C, G and U) and the seven dihedral angles *α*, *β*, *γ*, *χ*, *δ*, 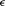 and 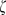 (Figure 1). In many other parameterizations, one or more additional parameters are used, such as the dihedral angles in the sugar ring [22]. However, it is possible to calculate the positions of all non-hydrogen atoms in an RNA structure based on the seven dihedral angles and the base type using the SN-NeRF algorithm [24] and assuming ideal bond lengths and angles [25]. This parameterization is advantageous as it is simple, yet sufficient to describe any RNA conformation in atomic detail on a local length scale.

### Description of the probabilistic model

The aim of the model, BARNACLE (BAyesian network model of RNA using Circular distributions and maximum Likelihood Estimation), is to capture both the marginal distributions of each of the seven angles and the local dependencies between them. The main ideas behind the design of the model are (i) to model the marginal distributions of the seven dihedral angles by mixtures of univariate probability distributions, since such mixtures have proven ideal for approximating arbitrary distributions [26], and (ii) to model the dependencies between the angles through a Markov chain of hidden states.

**Figure 2:**
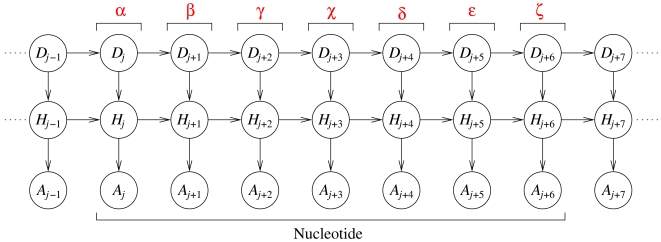
BARNACLE: a dynamic Bayesian network (DBN) that models the dihedral angles in an RNA fragment. In the graph, the nodes represent stochastic variables, and the arrows encode their conditional independencies. That is, the graph structure specifies the form of the joint probability distribution of the variables. The shown DBN represents nine consecutive dihedral angles, where the seven central angles originate from a single nucleotide. Each slice 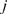 (a column of three variables) corresponds to one dihedral angle in an RNA fragment. The variables in each slice are: an angle identifier, *D_j_*, a hidden variable, *H_j_*, and an angular variable, *A_j_*. The angle identifier keeps track of which dihedral angle (from 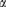 to 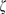) is represented by a slice, while the angular node models the actual dihedral angle value. The hidden nodes induce dependencies between all angles along the sequence (and not just between angles in consecutive slices).

We have implemented these ideas in a DBN (Figure 2) that uses one slice (with position index 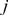) for each angle in the parameterization of a given RNA fragment. For example, for two nucleotides 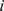 and *i*+1, the DBN consists of 14 slices that represent the angles
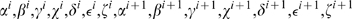
in the given order. Each slice, *j*, consists of three stochastic variables: an angle identifier, *D_j_*, that specifies which of the seven angles is represented in a given slice, a hidden variable, *H_j_*, that can adopt 20 different discrete states (which is the optimal number of states, see below and Materials and Methods), and an angular variable, *A_j_*, that adopts values in the interval 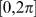. The DBN models the conformational space of an RNA molecule with 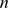 angles by the probability distribution:
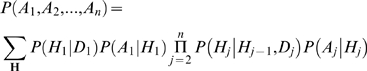
(1)where the sum runs over all possible hidden node sequences 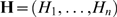.

We model all the factors in this expression that involve discrete variables as conditional probability tables. To model the angular variable, we use the univariate von Mises distribution [27]. This is the circular equivalent of the Gaussian distribution, with the density function
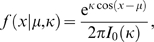
where 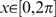 is the angle, 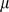 is the mean angle, 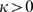 is a concentration parameter and 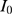 is the modified Bessel function of the first kind, order 0. More precisely, we use a von Mises distribution to model each of the 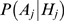 distributions, with parameters determined by the value of the *H_j_*. In this way each dihedral angle distribution is modeled as a weighted sum over the same set of 20 von Mises distributions. This idea is crucial for the development of a tractable model of this high dimensional space, as it leads to a very economical model, in which many parameters are common. Only 40 parameters are used for the von Mises distributions, which represent the angles in continuous space. The final model has only 537 non-zero parameters.

All the parameters are estimated by maximum-likelihood estimation from experimental RNA data (see Materials and Methods). The calculation of the sum in the probability density function (equation (1)) can be efficiently calculated using the *forward algorithm* [28]. Also, efficient algorithms exist to sample from the probability distribution (see Materials and Methods).

We use the base type information in the construction of the 3-D atom positions, but do not explicitly represent the base type in the probabilistic model. The model only includes dihedral angles, and is thus a purely geometrical model. The reasons not to include base information directly into the model are two-fold: (i) by focusing on a purely geometric model we diminish the dimensionality of the problem, which is already substantial relative to the amount of data available, and (ii) the geometric model can easily be augmented with base information by a suitable energy function since the parameterization allows for the positioning of all the atoms in the base.

### Evaluation of BARNACLE

In the following section, we evaluate the model using four tests. In the first two tests, we examine how well the model describes local RNA structure by (i) an information-theoretic analysis of the angular distributions, including the distributions of individual angles and pairs of angles, and (ii) analyzing the length distribution of the most abundant substructure in RNA, the A-helix. In the third test, we examine if the model is consistent with the rotamer model introduced by Murray *et al.* [9]. Finally, in the fourth test we evaluate how well the model performs in an MCMC algorithm for global RNA 3-D structure prediction. In the first three tests, we use a standard data set of experimentally determined RNA structures [9].

To the best of our knowledge, this is the first probabilistic model of local RNA 3-D structure in continuous space. Therefore, we construct our own baseline model for a meaningful comparison. The baseline model has the same design as BARNACLE (Figure 2), but without the (horizontal) arrows between the hidden variables, thereby removing the dependencies along the sequence. Such a model is called a *mixture model*. The use of a mixture model as baseline is highly appropriate for two reasons. First, a mixture model is theoretically able to approximate the marginal distributions of the individual angles arbitrarily well [26], and thus constitutes a challenging baseline. Second, it gives us the opportunity to test to what extent BARNACLE benefits from including sequential dependencies.

### Information-theoretic analysis of BARNACLE

In the first test, we compare BARNACLE to the mixture model using the information-theoretic approach, following Burnham and Anderson [29]. This approach is based on the Kullback-Leibler (KL) divergence, which is a natural measure of the distance (expressed in bits) between probability distributions [30]. For the selection of the best model for a given data set, this leads to the use of the Akaike Information Criterion (AIC). The AIC reaches a minimum value for the best model.

For BARNACLE, the minimum AIC value is reached at 20 hidden states and for the mixture model, at 25 hidden states (Materials and Methods). According to the minimum AIC values, BARNACLE clearly outperforms the mixture model as a joint distribution over the data set, which illustrates the importance of taking the sequential dependencies into account.

**Figure 3:**
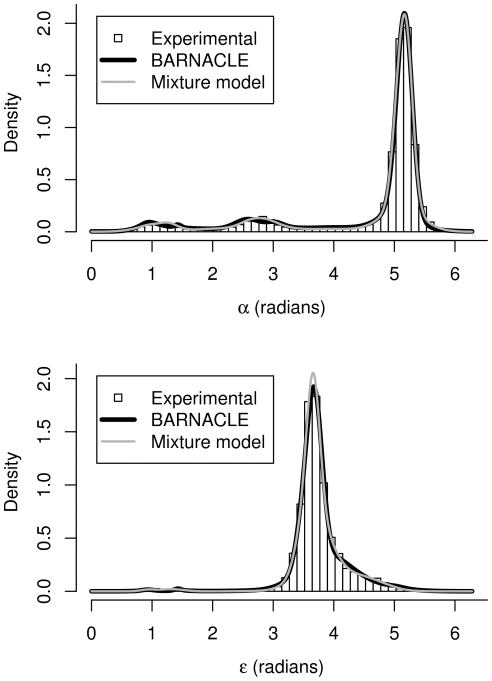
The distributions of the 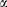 and 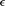 angles. The top figure shows the distributions of the 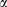 angle and the bottom figure shows the distributions of the 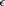 angle. The distributions in the experimental data set are shown as histograms. The density functions for the angles in the mixture model and BARNACLE are shown as light and dark grey lines, respectively. Both models capture the tri-modal nature of the 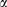 angle and the skewed distribution of the 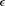 angle. See Figure S2 for plots of all 7 angles.

**Figure 4:**
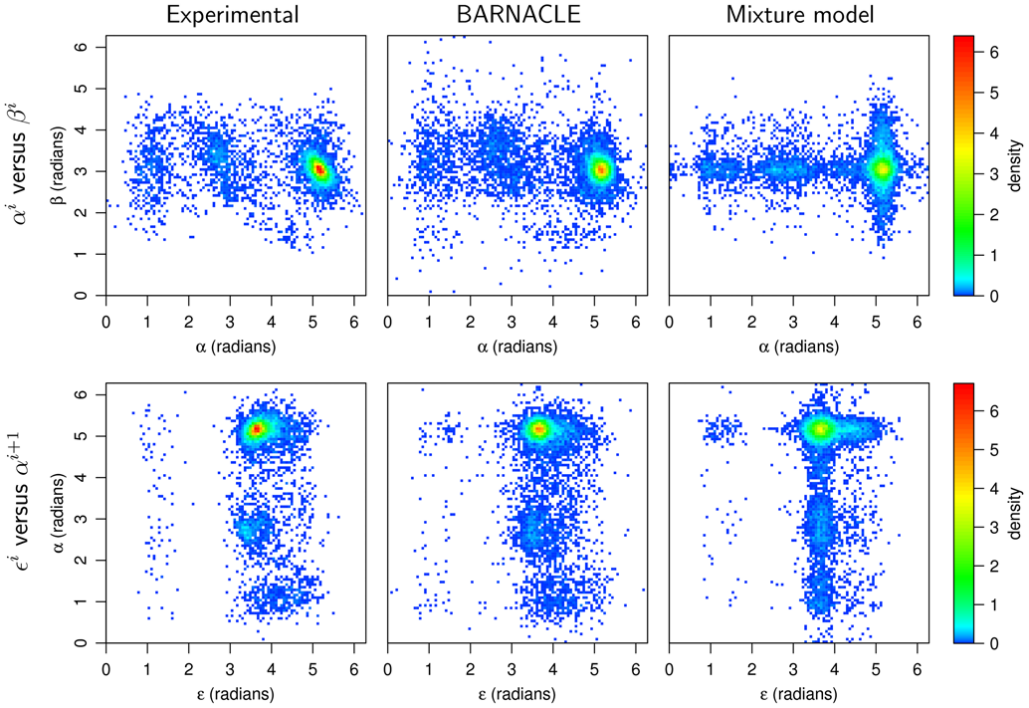
Histograms of pairwise angle distributions. The figure shows the distributions in the experimental data set (left column) and in data sampled from BARNACLE (middle column) and the mixture model (right column). Top row: the pairwise distributions of the dihedral angles 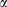 and 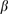 within a nucleotide. Bottom row: the pairwise distributions of the inter-nucleotide angles 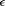 and *α*, where each 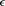 angle is paired with the neighboring 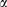 angle in the 3′-end direction.

Both models capture the multimodal nature and the skewness of the marginal distributions of the seven individual angles (Figure 3 and Figure S1). The mixture model is expected to be more accurate at the level of the individual angular distributions [26], since sequential restraints are absent during its estimation. A comparison based on the difference between the KL divergence of the two models to the experimental data shows that this is indeed the case (see Table S1). This fact establishes the mixture model as a challenging baseline. However, the superiority of BARNACLE already becomes clear at the level of the pairwise angular distributions (within the same nucleotide, Table S2A, and in consecutive nucleotides, Table S2B). The difference in accuracy between the two models is also clearly visible in the corresponding pairwise histograms (Figure 4 and Figure S2).

### BARNACLE captures the length distribution of helices

In the second test, we evaluate how well BARNACLE captures the length distribution of the helical regions in RNA. The idea is to examine how well BARNACLE captures longer range dependencies between the dihedral angles. We do so by first sampling a set of structures from both BARNACLE and the mixture model (see Materials and Methods). We then use the publicly available program Suitename [31] to identify all A-helix rotamers in both the sampled data sets and in the experimental data set. Finally, we analyze the distributions of the helix lengths in the three data sets, where helix length is defined as the number of consecutive A-helix rotamers.

**Figure 5:**
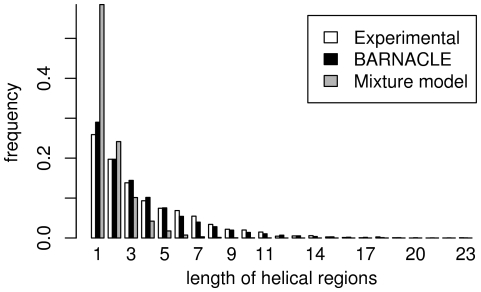
Histograms of the lengths of helical regions. The distributions in the experimental data set, and in the data sets sampled from BARNACLE and the mixture model are shown. The length is defined as the number of consecutive A-helix rotamers.

The histograms for the experimental data set and the data set sampled from BARNACLE exhibit the same exponentially decaying distribution (Figure 5). In contrast, the histogram for the samples drawn from the mixture model decays significantly faster than the two others. The differences can again be quantified using the KL divergence. For the histograms of helices up to length 16, the KL divergence from the experimental length distribution to the length distribution in the BARNACLE data set is 0.014 bits, whereas the KL divergence for the mixture model data set is as large as 1.10 bits.

In conclusion, BARNACLE captures the length distribution of the helical regions. The comparison with the mixture model makes it clear that in this context the model benefits considerably from including the sequential dependencies between the angles.

### BARNACLE is consistent with an established rotamer model

In the third test, we evaluate whether BARNACLE is consistent with a discrete rotamer model that was first introduced in 2003 by Murray *et al.* [9]. This rotamer model is currently used in the software package MolProbity [12] for validation of the local structure of experimentally determined structures. In this model, all local structures are clustered into 46 different types, each represented by a single rotamer.

We first sample a set of structures from BARNACLE and the mixture model (see Materials and Methods). The rotamers in the sampled and the experimental data sets are categorized using the program Suitename [31], and their frequencies of occurrence are compared.

**Figure 6:**
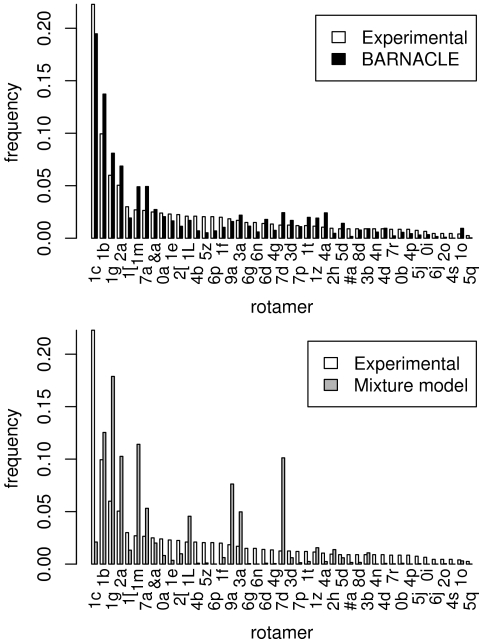
Histograms of the rotamer distributions in the non-helical regions. The figure shows the distributions in the experimental data set (top and bottom), in the BARNACLE samples (top) and in the mixture model samples (bottom). The names of the rotamers, as defined by the RNA Ontology Consortium [31], are used as index on the horizontal axis. The rotamers are sorted along the horizontal axis according to their frequency in the experimental data set.

Strikingly, all 46 rotamer types are present in the BARNACLE samples. In addition, the fractions of the 45 non-helical rotamer types are similar in the experimental data set and in the BARNACLE samples (Figure 6). Finally, the percentage of A-helix rotamers also matches closely (74.6% in the experimental data set and 76.1% in the BARNACLE data).

Turning to the mixture model for comparison, we see that the fractions of the 45 non-helical rotamers in the experimental data set and in the samples are markedly different (Figure 6), and that the percentage of A-helix rotamers is considerably lower than in the experimental data set (53.2% versus 74.6%). In addition, the percentage of conformations that do not belong to any of the rotamers is markedly higher for the mixture model (28.0%) than for BARNACLE (20.1%) and the experimental data set (14.2%). Finally, the KL divergence from the distribution of the 46 rotamers in the experimental data set is higher to the mixture model data (1.83 bits) than to the BARNACLE data (0.20 bits).

Hence, BARNACLE is consistent with the rotamer model and also in this context, the model benefits from including sequential dependencies.

### BARNACLE generates RNA-like decoys

In the fourth and final test, we use BARNACLE to generate decoy structures for ten different RNA target structures, solely using coarse-grained base-pairing information (that is, secondary structure information).

We generate these structures using an MCMC method based on 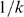 multihistogram sampling [32],[33], which makes it possible to obtain samples from BARNACLE that fall within a specified, favorable energy interval. In other words, we can sample from BARNACLE conditional upon a favorable energy. As energy function, we use a simple base pairing energy (measured in Å) that reaches a minimum when all the hydrogen bonds that are implied in the native secondary structure are present. In this way, we sample a large number of structures with correct secondary structure, but with all the fine-grained conformational details entirely left up to BARNACLE. The goal of this test is to examine whether BARNACLE is capable of generating plausible RNA structures from coarse grained base-pairing information only.

The test consists of using the MCMC method to generate a large number of decoys for each of the ten targets (see Table S3 for details on execution). We consider all decoys that have good secondary structure (energy less than 1.0 Å) and evaluate their *all-atom RMSD* (including all non-hydrogen atoms) and the *C4′ trace RMSD* after optimal superimposition with the target RNA structure.

As a baseline, we again use the mixture model. We also include another baseline; a model in which each angle distribution is modeled by the uniform distribution on the circle. The RMSD values for the best decoys are shown in Table 1. In this table, we have for comparison also included results from the lowest RMSD decoys obtained by Das and Baker's FARNA method on the same set of structures [14]. The target structures we use in this test are the single chain subset of the structures used to evaluate FARNA. To avoid bias, the models were re-trained on structures that were not homologous to any of the target structures [14] (see Materials and Methods).

**Table 1:** Generation of RNA decoys using secondary structure information.

Structure description			BARNACLE		Mixture model		Uniform model		FARNA
PDB ID	Len	Bps	RMSD	C4′ RMSD	RMSD	C4′ RMSD	RMSD	C4′ RMSD	C4′ RMSD
1ESY	19	6	**2.44**	**1.26**	2.61	1.43	8.14	6.96	1.44
1KKA	17	6	**2.97**	2.23	3.45	2.16	6.57	5.42	**2.08**
1L2X	27	8	**3.87**	**2.77**	4.99	4.02	9.11	8.28	3.11
1Q9A	27	6	**3.35**	2.92	5.01	4.41	8.70	7.82	**2.65**
1QWA	21	8	**2.96**	2.26	3.33	2.60	7.75	7.46	**2.01**
1XJR	46	15	**9.50**	9.36	-	-	-	-	**6.25**
1ZIH	12	4	**0.95**	**0.80**	1.30	0.82	5.64	4.27	1.03
28SP	28	8	**2.52**	**2.10**	5.53	4.70	9.97	9.79	2.31
2A43	26	7	**3.58**	**2.65**	4.84	3.73	10.23	9.23	2.79
2F88	34	13	**3.00**	**2.35**	5.11	4.78	-	-	2.41

*Len*: the number of nucleotides in the molecule; *Bps*: the number of Watson Crick and G–U wobble base pairs in the structure; *RMSD*: the all-atom RMSD of the decoys with the lowest all-atom RMSD from the native structure; *C4′ RMSD*: the C4′ RMSD of the decoy with the lowest C4′ RMSD from the native structure. *FARNA C4′ RMSD*: the C4′ RMSD for the decoys with the lowest C4′ RMSD obtained by Das and Baker's FARNA method [14]. A dash indicates that no structures with good base paring (energy below 1.0 Å) were obtained. All RMSD values are measured in Ångström (Å). Lowest (best) RMSD values are highlighted with bold face.

As shown in Table 1, BARNACLE generates good decoys for all but the longest of the target structures (1XJR, which is equally challenging for the FARNA method). Most of the best decoys have all atom RMSD values below 4 Å, and C4′ RMSD values below 3 Å, and are thus close to the native target structure [14]. In all but one case, the best BARNACLE decoys have a lower RMSD than the best decoys generated using the mixture model, while in all cases, the uniform model performs considerably worse. The mixture model performs surprisingly well; for some of the targets the best decoys have an all-atom RMSD that is below 3 Å. However, when considering the RMSD distribution of all sampled decoys with good secondary structure, we see that BARNACLE generates more low RMSD decoys than the mixture model (Table S4); the 25% RMSD quantile for BARNACLE is in general lower than or about equal to the 5% quantile for the mixture model.

**Figure 7:**
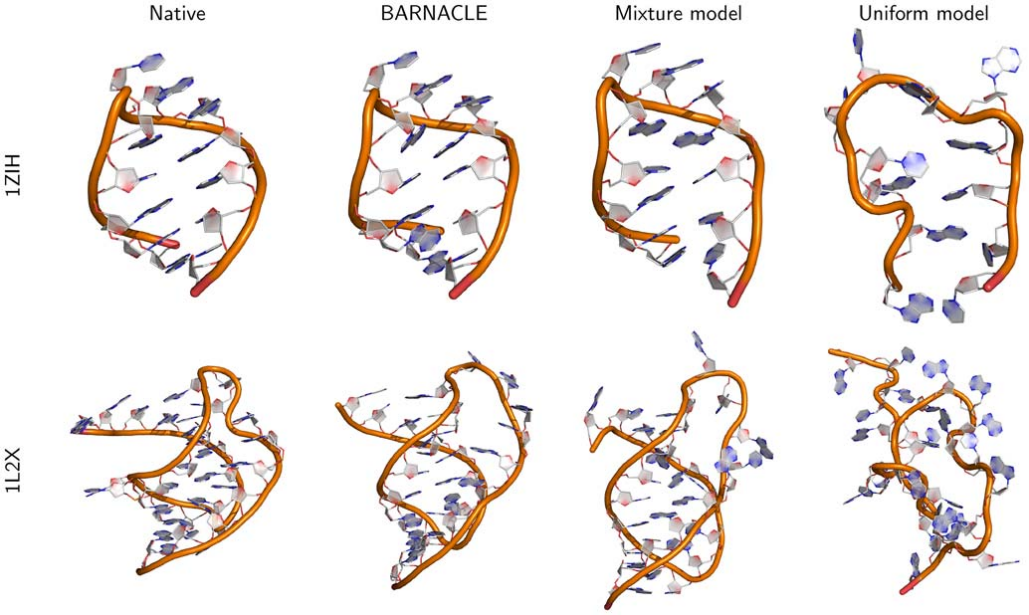
Decoys generated using BARNACLE, the mixture model and the uniform model. The decoys shown are those with the lowest full-atom RMSD from the native structures, among all decoys with good secondary structure (energy less than 1.0 Å). Decoys are shown for PDB structures 1ZIH and 1L2X. Pictures made using PyMOL (http://www.pymol.org).

The best decoys for 1ZIH and 1L2X are shown in Figure 7. Note that for the structures generated with BARNACLE, the course of the backbone is very close to the native, and that for 1ZIH all the bases in the challenging loop region are also placed correctly. This can only be ascribed to the model, as the correct conformation of the backbone and of the bases in the loop is not directly promoted by the energy function. Figure 7 clearly illustrates another way in which BARNACLE outperforms the mixture model: in the case of 1L2X, the course of the backbone is less RNA-like for the mixture model. These qualitative observations are confirmed quantitatively by the “suiteness” score (a structural quality score [12]) of the decoys, which shows a considerably lower quality for the mixture model decoys (Table 2). The uniform model performs much worse than both BARNACLE and the mixture model. Essentially it does not produce any realistic RNA conformations.

**Table 2:** The average suiteness scores for the lowest RMSD decoys.

Structure description			BARNACLE		Mixture model		Uniform model		Target
PDB ID	Len	Bps	Best struct	Best C4′ struct	Best struct	Best C4′ struct	Best struct	Best C4′ struct	
1ESY	19	6	**0.755**	**0.786**	0.571	0.640	0.000	0.000	0.168
1KKA	17	6	**0.756**	**0.737**	0.715	0.637	0.000	0.000	0.210
1L2X	27	8	**0.652**	**0.629**	0.619	0.566	0.000	0.000	0.745
1Q9A	27	6	**0.731**	**0.705**	0.604	0.575	0.001	0.000	0.714
1QWA	21	8	**0.789**	**0.827**	0.722	0.723	0.000	0.000	0.077
1XJR	46	15	**0.706**	**0.706**	-	-	-	-	0.508
1ZIH	12	4	**0.784**	**0.784**	0.675	0.610	0.000	0.000	0.505
28SP	28	8	**0.812**	**0.816**	0.601	0.589	0.000	0.000	0.328
2A43	26	7	**0.664**	**0.675**	0.529	0.456	0.000	0.000	0.692
2F88	34	13	**0.746**	**0.776**	0.556	0.557	-	-	0.509
Average suiteness			**0.732**	**0.737**	0.610	0.585	0.000	0.000	0.497

The table shows the average scores for the lowest RMSD decoys generated by BARNACLE, the mixture model and the uniform model. The average scores are calculated by Suitename [31] and higher scores indicate higher quality. *Len*: the number of nucleotides in the molecule; *Bps*: the number of Watson Crick and G–U wobble base pairs in the structure; *Best struct*: the average suiteness per suite for the lowest RMSD structure; *Best C4′ struct*: the average suiteness per suite for the lowest C4′ RMSD structure; *Target*: the average suiteness per suite for the experimental determined target structures. The highest (best) suiteness scores are highlighted with bold face. A dash indicates that no structures with the correct base paring (energy below 1.0 Å) were obtained.

It is finally worth noticing that the results obtained with BARNACLE for the ten structures are comparable to the results obtained with the FARNA method by Das and Baker [14]; for 6 of the target structures BARNACLE generates decoys with a lower RMSD than FARNA. BARNACLE (a sampling method, which we combine here with a very simple energy function based on native secondary structure) and FARNA (a full blown RNA prediction method) are of course very different methods, but the results indicate that BARNACLE can be used to generate state-of-the-art decoys in the context of 3-D RNA structure prediction in atomic detail.

However, the crucial improvement introduced by BARNACLE lies in providing a fully probabilistic sampling framework in continuous space, while maintaining state-of-the-art sampling quality (as shown by the comparison with FARNA). As pointed out before, sampling methods based on fragment assembly impose serious limits on the form of the energy function, and necessitates the use of unphysical energy terms. BARNACLE provides a satisfactory solution to this problem. The potential importance of BARNACLE is also illustrated by the enormous impact of the introduction of rigorous sampling methods on RNA secondary structure prediction [4],[5].

## Discussion

This study introduces a new approach to modeling local RNA 3-D structure. In contrast to previous approaches, we model the local conformational space as continuous, and in a fully probabilistic framework.

The introduced model has the potential to improve current structure prediction approaches in several ways. First, it allows for continuous adjustments in the conformational space, which accommodates the use of fine-grained energy terms. As pointed out by Das and Baker [14], discrete models preclude that. Second, the probabilistic nature of BARNACLE enables unbiased sampling in an MCMC framework and makes it possible to include the local structural bias as a direct term in an energy function, which is not possible with non-probabilistic models.

Our model has several other potential uses, such as RNA structure validation. The current state of the art is to assign scores to short individual fragments based on their similarity to a set of rotamers [12]. The model proposed here could be used to assign a likelihood to a whole sequence of dihedral angles or to pinpoint local stretches that have a low likelihood.

As for the quality of the model, we have shown that it captures the essential properties of local RNA structure, and that it is consistent with the rotameric model of RNA that underlies the structure validation tool MolProbity [12]. In addition, we have demonstrated that the model readily generates good quality decoys for short RNA molecules using an MCMC framework and a simple energy function.

An obvious challenge for the future is to extend the model with sequence and evolutionary information. Given the high dimensionality of the problem, and the paucity of the data, this will pose a formidable statistical challenge.

With the development of the probabilistic model of local RNA structure and our previous work on probabilistic models of local protein structure [20],[21], we have provided solutions to the conformational sampling problem for the two most important biological macromolecules: RNA and proteins. We expect to see considerable benefits from these models in many areas of application.

## Materials and Methods

### Training and selecting a model

To obtain the final model, we optimized BARNACLE's parameters based on a set of known RNA structures, using the in-house dynamic Bayesian network software package Mocapy [34]. The optimization was done with the *stochastic expectation maximization* algorithm [35].

#### Selecting number of hidden states

The optimal number of hidden states for BARNACLE was determined using the Akaike Information Criterion (AIC). We chose AIC over the two other model selection criteria, the *Bayesian Information Criterion* and the *Integrated Completed Likelihood*, since both criteria are known to underestimate the number of hidden states for density estimation [36],[37], and clearly do so for our particular model design (data not shown). Both criteria point to models with fewer hidden states than the total number of modes in the angle distributions.

**Figure 8:**
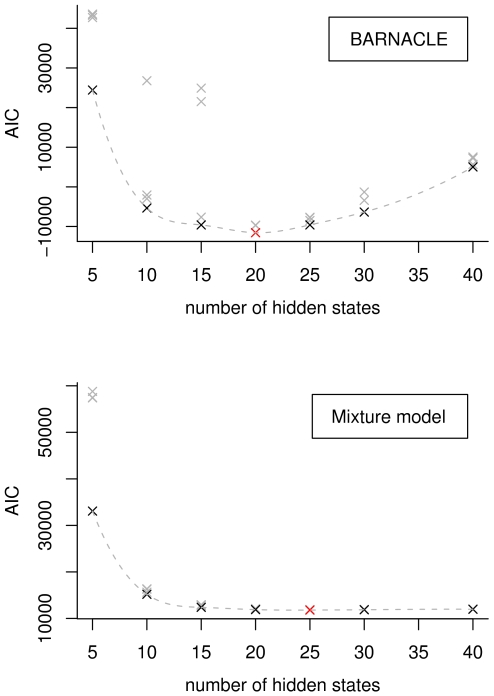
Selection of the best models using the Akaike Information Criterion. The Akaike Information Criterion (AIC) scores are shown for all trained BARNACLE models (top) and mixture models (bottom). The AIC score reaches a minimum for the best model. The BARNACLE model with 20 hidden states, and the mixture model with 25 states have the best AIC scores (shown in red). The best models for each given number of hidden states are shown in black. The dotted lines are tendency lines constructed by cubic splines [43]. The three outliers in the BARNACLE plot (at 10 and 15 hidden states) illustrate that the stochastic expectation maximization procedure can get stuck in a local optimum. Note that the best BARNACLE model has a lower (better) score than the best mixture model.

We trained seven models with 5, 10, 15, 20, 25, 30 and 40 hidden states, respectively. Each of these models were trained with 4 different initial sets of parameters, to avoid picking a model that converged to a local optimum. We chose the model with the lowest AIC score, resulting in 20 hidden states. We used the same training procedure for the baseline mixture model, which resulted in a model with 25 hidden states. The AIC plots for the two models can be seen in Figure 8.

#### The Akaike Information Criterion

The Akaike Information Criterion (AIC) [26],[29] is a well established model selection criterion that favors the model which minimizes the expression
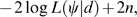
where 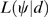 is the likelihood of the model 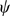 given the data *d*, and 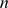 is the number of parameters. The AIC score is an estimate of the expected relative Kullback-Leibler divergence between the unknown mechanism that generated the data and the model fitted to the data [29] (for a definition of Kullback-Leibler divergence see below).

### Training data

As training data, we used the angles from the structures in the 2005 version of the RNA data set compiled by Murray *et al.* [9], which consists of RNA 3-D structures of good quality determined by X-ray crystallography. For all the tests, except the decoy test, we used the entire data set for training. For the decoy test, we trained the models using the RNA data in the large ribosomal subunit (PDB code 1S72) in order to avoid bias from homologous structures [14]. For target 1Q9A, the homologous sequence at residues 2684–2710 in structure 1S72 was removed before training. Before we used the data set we removed outliers and ensured that the data consists of unbroken chains.

#### Outlier removal

The compilers of the data set pointed out that the data set contains errors [9]. Hence, we performed an outlier removal by applying the outlier definition of Knorr and Ng [38] to every angle pair within a residue. This led to the removal of the worst outliers, but did not significantly decrease the size of the data set: 971 out of 70,803 angles were removed.

#### Chain breaks

Some PDB files in the data set lack whole residues in the middle of a chain. We identified such residues by considering the bond distances O3′-P between consecutive residues. When such a distance was more than 50 times the standard deviation [25], we split the chain up at this point. Since we want to preserve the sequentiality in the data set, we use the Needlemann-Wunch algorithm [39] to align all the pieces to the full base sequence specified in the PDB header (the algorithm was modified to only allow insertions at split points). In this way, we calculate how many residues are missing. The missing residues are simply treated as missing data in the stochastic expectation maximization training procedure [35].

### Sampling

It is possible to sample from BARNACLE in two different ways: one can (i) sample an entire sequence of angles, or (ii) resample a segment in an angle sequence seamlessly, that is, conditional upon the remaining angles. In both cases, the resulting angle sequence is subsequently converted into atomic coordinates.

#### Sampling a sequence of angles

Sampling a sequence of angles is done using a three step procedure. First, one specifies the values of the angle identifier nodes, which for an RNA fragment of 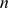 nucleotides consists of 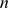 repeats of the sequence *α*, *β*, *γ*, *χ*, *δ*, 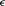 and *ζ*. Then, the values of the hidden nodes, 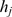 are sampled from one end to the other, from the distribution 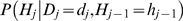. Finally, the angular values are sampled from the distribution 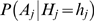.

#### Resampling a segment of angles

Assume that we have sampled a sequence of hidden values, 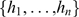, and a sequence of angle values, 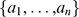, given an appropriate sequence of identifier variables, 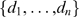. Resampling a subsequence, from position 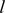 to 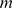 can then be done using the forward-backtrack algorithm [20],[21],[40]. This algorithm is a two step procedure.

In the first step the hidden variables, 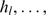 *h_m_*, are resampled. This is done by first calculating the forward variables
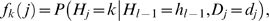
for each possible hidden node value 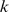 in each slice 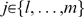, using the forward algorithm [28]. Then the hidden nodes values, 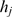, are sampled from position 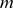 to position 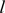 proportional to:
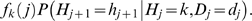
In the second step the angles, 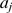, at each position 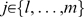, are sampled from the distribution 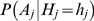.

### Data sets used in the evaluations

We use data sets sampled from BARNACLE and the mixture model for the evaluations in the results section.

For the comparison of the pairwise angle distributions (Figure 4 and Figure S2) we sampled data sets with the same size (9.8⋅10^3^ nucleotides) and length distributions as the experimental data set.

For the comparison of the length distributions of helical regions (Figure 5) and the rotamer distribution (Figure 6), we sampled data sets of 100 times the size of the experimental data set (0.98⋅10^6^ nucleotides), again with the same length distributions as the experimental data set. For these two comparisons, the data set has to be this large to ensure a sufficient sampling of the distribution in question from the mixture model. Certain rotamers (Figure 6) and long helical regions (Figure 5) only have negligible probabilities according to the mixture model, and for smaller sample sizes not all rotamers are sampled.

### Model evaluation using the Kullback-Leibler divergence

In the Results section, we use the Kullback-Leibler (KL) divergence [30] to measure the similarity between the experimental data and the models.

The KL divergence is a standard measure for the distance between two probability distributions. For two continuous probability density functions 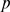 and 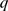, the KL divergence is defined as:
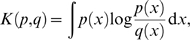
(2)while for two discrete probability mass functions 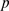 and 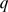 the KL divergence is defined as
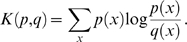
(3)Typically 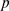 is taken to be an empirical data distribution or the “true” underlying distribution that generated the data, whereas 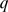 typically represents a model or an approximation of *p*. The divergence is always non-negative and only becomes zero for equal distributions. When the binary logarithm is used in the definition, the divergence is measured in bits.

For the comparisons of the individual and pairwise angle distributions we use equation (2). We calculate the difference between the KL divergence from the experimental data set, *p*, to the mixture model, 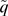, and the KL divergence from the experimental data set to BARNACLE, *q*, in the following way:
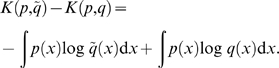
To calculate this expression, we use the fact that the KL divergence can be expressed in terms of statistical expectations [29]. The difference can be rewritten as the expectation with respect to 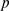:
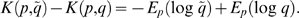
Since the empirical distribution, 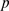, is a set of observations, 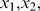 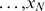, we can calculate the expectations by averaging over these observations [41]:
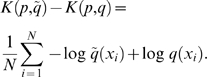



For the length distribution of the helical regions and the distribution of the 46 rotamers we use equation (3).

### Details on the MCMC simulations

The MCMC simulations are done in the 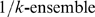 [32], using the Metropolis-Hastings algorithm [17],[42], and the generalized multihistogram method for updating the weights [33]. The method has two main components: a proposal distribution, and an energy function (see below for details). The energy space is divided into 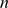 bins (each of width 0.05 Å), and the method seeks to generate samples more often in low than in high energy bins. In particular, the target distribution is the density of states weighted according to the inverse of the cumulative density of states [33]. The final ensemble of sampled structure has the approximate property that the distribution of samples within each energy bin is the proposal distribution. In other words, we generate samples from BARNACLE that are conditional upon belonging to a low energy bin.

#### Proposal distribution

We use three different models (BARNACLE, the mixture model and the uniform model) for the proposal distributions. For all three models, the proposals are constructed in the following way.

Let 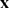 be the current candidate structure with the angle sequence **x** *_a_*. The next candidate structure, **x′**, is then proposed by resampling a stretch of angles in 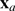 according to the model, and calculating the atom positions corresponding to the new angle sequence **x′** *_a_*. For BARNACLE the resampling is efficiently done using the forward-backtrack algorithm (for a description see section on Sampling). For the mixture model and the uniform model, each angle in the subsequence can be resampled individually, since all angles are independent according to these models.

The length of the sequence to be resampled is drawn from a Poisson distribution with mean 2 that is truncated at the maximum number of angles in the target structure.

We require that all sampled structures are clash free; if a clash occurs, the structure is immediately rejected. We define a clash as a pair of non-covalently bonded atoms that are closer to each other than 1.8 Å.

#### Energy function

We use a distance-based energy function that enforces a desired secondary structure (Watson Crick and G–U wobble base pairs). The energy function is constructed in the following way.

Let 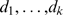 be the distances between the donors and acceptors in each of the hydrogen bonds making up the desired secondary structure in a structure 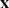 (every A–U and G–U pair contributes two distances, and every C–G pair contributes three distances). The base paring energy of 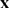 is then defined as
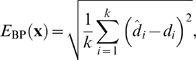
where 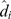 is a reference value for the hydrogen bond distance in the particular type of base pair. The reference value for each of the 7 donor-acceptor distances is calculated as the mean distance in the structures from the 2005 version of the RNA data set compiled by Murray *et al.* [9]. The energy is measured in Å, and the minimal base pair energy of 0 Å is only obtained for structures with perfect base paring.

For the simulations presented in Table 1, the enforced secondary structure is the secondary structure of the target structure.

### Availability

A software implementation of BARNACLE is freely available on SourceForge (http://sourceforge.net/projects/barnacle-rna/).

## Supporting Information

Figure S1 The marginal distributions of all seven individual angles.
(0.06 MB PDF)

Figure S2 Histograms of pairwise angle distributions with the highest and lowest KL difference.
(0.49 MB PDF)

Table S1 The KL divergences for the seven individual angles.
(0.01 MB PDF)

Table S2 The KL divergences for angle pairs.
(0.02 MB PDF)

Table S3 Execution time of the MCMC algorithm.
(0.02 MB PDF)

Table S4 The 5% and 25% quantiles of the RMSD distributions for decoys with correct base pairing.
(0.02 MB PDF)
