# Supplementary material for: A Probabilistic Model of RNA Conformational Space
Source: PLoS Comput Biol. 2009 Jun 19;5(6):e1000406. doi: 10.1371/journal.pcbi.1000406 (PMC2691987; doi:10.1371/journal.pcbi.1000406)
Supplement: Figure S1 — The marginal distributions of all seven individual angles. (0.06 MB PDF) [file pcbi.1000406.s001.pdf]

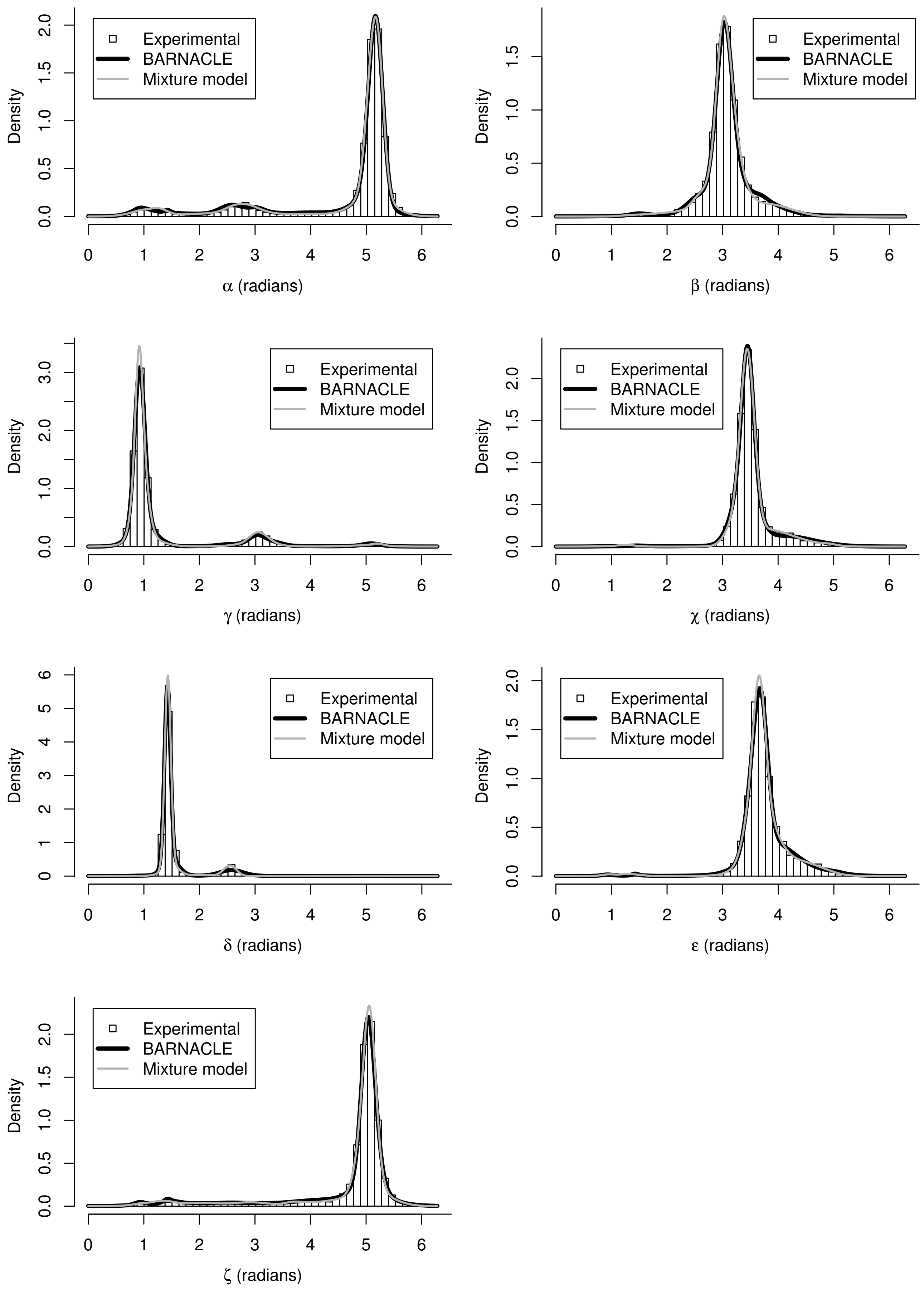


**Figure S1. The marginal distributions of all seven individual angles.** The distributions in the experimental data set are shown as histograms. The density functions for the mixture model are shown as gray lines. The black lines show the densities for BARNACLE, calculated from the stationary probability distributions [Cappé O, Moulines E, Ryden T (2005) Inference in hidden Markov models. New York: Springer. 654 p.].
