# Supplementary material for: A Probabilistic Model of RNA Conformational Space
Source: PLoS Comput Biol. 2009 Jun 19;5(6):e1000406. doi: 10.1371/journal.pcbi.1000406 (PMC2691987; doi:10.1371/journal.pcbi.1000406)
Supplement: Figure S2 — Histograms of pairwise angle distributions with the highest and lowest KL difference. (0.49 MB PDF) [file pcbi.1000406.s002.pdf]

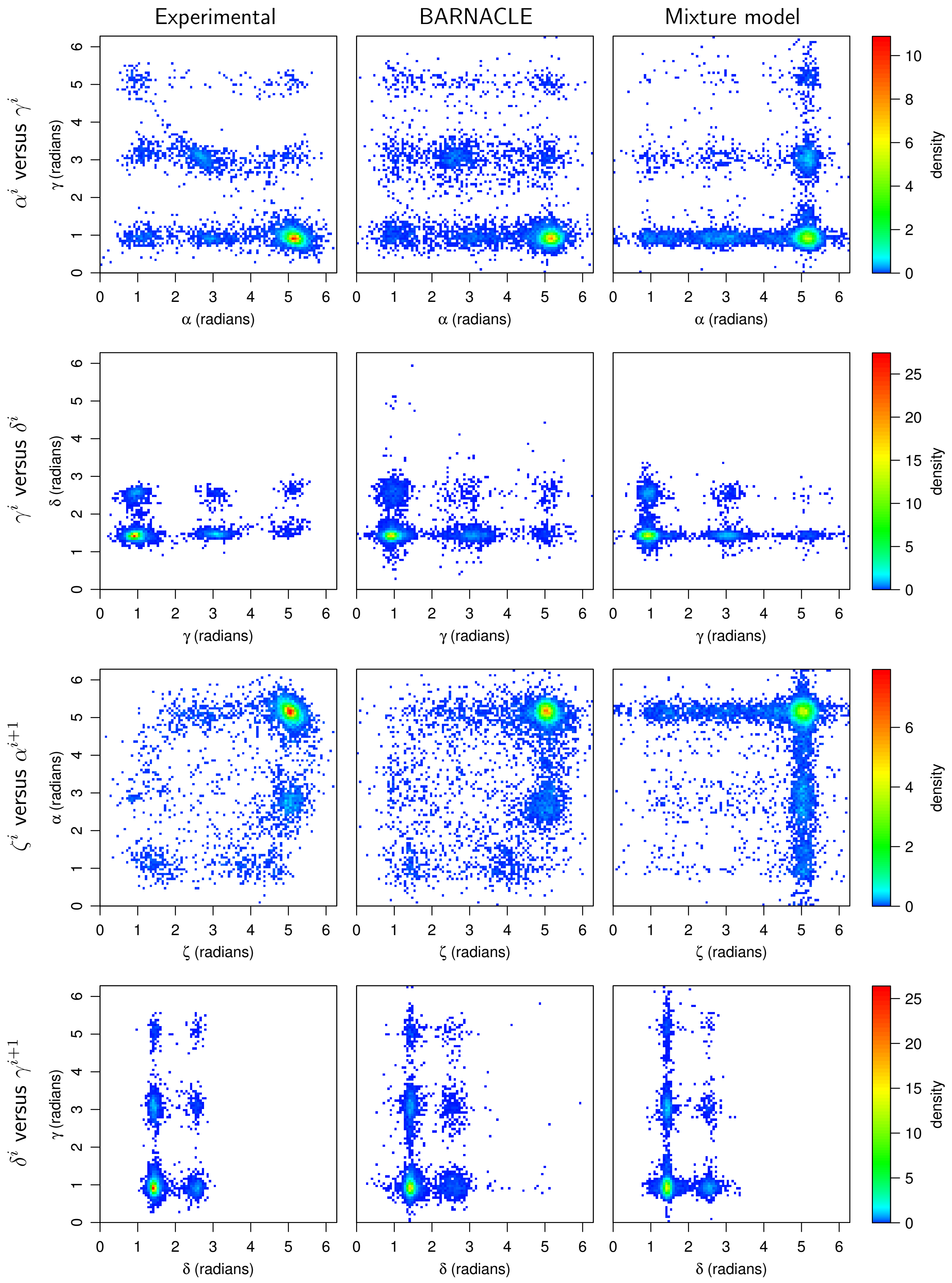

Figure S2. Histograms of pairwise angle distributions with the highest and lowest KL difference. The figure shows the pairwise angle distributions, where BARNACLE performs best (rows 1 and 3) and worst (rows 2 and 4), compared to the mixture model. Rows 1 and 2: The pairwise distributions *within nucleotides* with the highest KL difference (α^i versus γ^i at 0.301 bits) and the lowest KL difference (γ^i versus δ^i at -0.002 bits) from Table S2A. Rows 3 and 4: The pairwise distributions in *consecutive nucleotides* with the highest KL difference (ζ^i versus α^{i+1} at 0.279 bits) and the lowest KL difference (δ^i versus γ^{i+1} at -0.038 bits) from Table S2B.
