# Supplementary material for: A Probabilistic Model of RNA Conformational Space
Source: PLoS Comput Biol. 2009 Jun 19;5(6):e1000406. doi: 10.1371/journal.pcbi.1000406 (PMC2691987; doi:10.1371/journal.pcbi.1000406)
Supplement: Table S1 — The KL divergences for the seven individual angles. (0.01 MB PDF) [file pcbi.1000406.s003.pdf]

Table S1. The KL divergences for the seven individual angles.

α	β	γ	χ	δ	ϵ	ζ
-0.014	-0.013	-0.015	-0.016	-0.045	-0.017	-0.008

The table shows the KL divergence (in bits) from the experimental data to the mixture model, minus the KL divergence from the experimental data to BARNACLE, for the marginal distribution of the individual angles.
