# Supplementary material for: A Probabilistic Model of RNA Conformational Space
Source: PLoS Comput Biol. 2009 Jun 19;5(6):e1000406. doi: 10.1371/journal.pcbi.1000406 (PMC2691987; doi:10.1371/journal.pcbi.1000406)
Supplement: Table S2 — The KL divergences for angle pairs. (0.02 MB PDF) [file pcbi.1000406.s004.pdf]

Table S2. The KL divergences for angle pairs.

vs.	α^i	β^i	γ^i	χ^i	δ^i	ϵ^i	ζ^i
α^i	-	**0.267**	**0.301**	**0.134**	**0.018**	**0.056**	**0.023**
β^i	-	-	**0.177**	**0.098**	**0.001**	**0.039**	**0.028**
γ^i	-	-	-	**0.104**	-0.002	**0.021**	**0.004**
χ^i	-	-	-	-	**0.226**	**0.173**	**0.153**
δ^i	-	-	-	-	-	**0.172**	**0.120**
ϵ^i	-	-	-	-	-	-	**0.169**
ζ^i	-	-	-	-	-	-	-

(A)

vs.	α^{i+1}	β^{i+1}	γ^{i+1}	χ^{i+1}	δ^{i+1}	ϵ^{i+1}	ζ^{i+1}
α^i	-	-	-	-	-	-	-
β^i	-0.001	-	-	-	-	-	-
γ^i	-0.011	-0.019	-	-	-	-	-
χ^i	**0.073**	**0.057**	**0.004**	-	-	-	-
δ^i	**0.036**	-0.003	-0.038	-0.010	-	-	-
ϵ^i	**0.161**	**0.127**	**0.058**	**0.057**	-0.017	-	-
ζ^i	**0.279**	**0.174**	**0.053**	**0.093**	**0.024**	**0.041**	-

(B)

The table shows the KL divergence (in bits) from the experimental data to the mixture model, minus the KL divergence from the experimental data to BARNACLE for angle pairs. (A) The difference in KL divergence for all angle pairs *within nucleotides*. For 20 out of 21 angle pairs, BARNACLE is closer to the dataset than the mixture model. (B) The difference in KL divergence for all angle pairs up to 6 angles apart *in consecutive nucleotides*. For 14 out of 21 angle pairs, BARNACLE is closer to the dataset than the mixture model. Note that for consecutive nucleotides, the mixture model only becomes better for angles that are far apart. Bold face numbers indicate pairs where BARNACLE has a lower KL divergence from the dataset than the mixture model.
