# Supplementary material for: A Probabilistic Model of RNA Conformational Space
Source: PLoS Comput Biol. 2009 Jun 19;5(6):e1000406. doi: 10.1371/journal.pcbi.1000406 (PMC2691987; doi:10.1371/journal.pcbi.1000406)
Supplement: Table S3 — Execution time of the MCMC algorithm. (0.02 MB PDF) [file pcbi.1000406.s005.pdf]

Table S3. Execution time of the MCMC algorithm.

Structure description			BARNACLE	Mixture model	Uniform model	Number of
PDB ID	Len	Bps	Execution time (hours)	Execution time (hours)	Execution time (hours)	MCMC steps
1ESY	19	6	14.1	11.9	80.4	$11.8 \cdot 10^6$
1KKA	17	6	10.8	9.7	53.7	$10.6 \cdot 10^6$
1L2X	27	8	29.4	25.4	147.0	$16.9 \cdot 10^6$
1Q9A	27	6	27.2	20.6	92.9	$16.9 \cdot 10^6$
1QWA	21	8	23.4	15.3	76.8	$13.1 \cdot 10^6$
1XJR	46	15	185.5	147.3	2450.7	$57.6 \cdot 10^6$
1ZIH	12	4	8.8	4.8	20.0	$7.5 \cdot 10^6$
28SP	28	8	39.3	27.6	143.3	$17.5 \cdot 10^6$
2A43	26	7	24.0	23.6	108.2	$16.2 \cdot 10^6$
2F88	34	13	52.1	40.7	213.4	$21.2 \cdot 10^6$

The table shows the number of steps used in the MCMC algorithm and its executions time. *Len*: the number of nucleotides in the molecule; *Bps*: the number of Watson Crick and G-U wobble base pairs in the structure; *Execution time*: the execution time on a 2.6Ghz Intel Core 2 processor (using a single core) of a Python implementation of BARNACLE; *Number of MCMC steps*: the number of steps used in the MCMC algorithm.
