# Supplementary material for: A Probabilistic Model of RNA Conformational Space
Source: PLoS Comput Biol. 2009 Jun 19;5(6):e1000406. doi: 10.1371/journal.pcbi.1000406 (PMC2691987; doi:10.1371/journal.pcbi.1000406)
Supplement: Table S4 — The 5% and 25% quantiles of the RMSD distributions for decoys with correct base pairing. (0.02 MB PDF) [file pcbi.1000406.s006.pdf]

Table S4. The 5% and 25% quantiles of the RMSD distributions for decoys with correct base pairing.

Structure description			BARNACLE		Mixture model		Uniform model	
PDB ID	Len	Bps	5%	25%	5%	25%	5%	25%
1ESY	19	6	**2.99**	**3.28**	3.46	4.17	8.39	8.63
1KKA	17	6	4.40	5.02	**4.37**	**4.90**	8.14	8.52
1L2X	27	8	**5.43**	**6.88**	6.69	7.66	9.58	9.99
1Q9A	27	6	**4.80**	**5.42**	5.63	6.65	9.69	10.40
1QWA	21	8	**4.06**	**4.64**	4.24	4.81	8.11	8.53
1XJR	46	15	**10.41**	**11.01**	-	-	-	-
1ZIH	12	4	**1.72**	**2.16**	2.16	2.89	6.07	6.56
28SP	28	8	**3.23**	**3.76**	7.17	7.91	10.31	10.50
2A43	26	7	**4.72**	**6.08**	6.96	7.72	10.96	11.19
2F88	34	13	**3.82**	**4.41**	5.34	5.70	-	-

Len: the number of nucleotides in the molecule; *Bps*: the number of Watson Crick and G-U wobble base pairs in the structure; *5%*: the 5% quantiles measured in Å; *25%*: the 25% quantiles measured in Å. A dash indicates that no structures with correct base paring (energy below 1.0Å) were obtained. Lowest (best) RMSD values are highlighted with bold face.
